# The Relation between Residential Mobility and Internalizing and Externalizing Problems in Adolescence: The Role of Subjective Moving Experience, Gender, and Friendship Quality

**DOI:** 10.1007/s10964-024-02014-6

**Published:** 2024-05-24

**Authors:** Juul H. D. Henkens, Gonneke W. J. M. Stevens, Helga A. G. de Valk

**Affiliations:** 1 Netherlands Interdisciplinary Demographic Institute-KNAW/University of Groningen, The Hague, The Netherlands; 2 Department of Interdisciplinary Social Science, Utrecht University, Utrecht, The Netherlands

**Keywords:** Internalizing and externalizing problems, Residential mobility, Adolescence, Gender, Friendship quality

## Abstract

Adolescent residential mobility can be a stressful life event, potentially aggravating internalizing or externalizing problems. However, the longitudinal effects of residential mobility are understudied and may be context-dependent. This study investigates the longitudinal associations between adolescent residential mobility and internalizing and externalizing problems. Additionally, this study examines for whom residential moves are most detrimental by including subjective moving experience, gender, and friendship quality before the move as moderators. Longitudinal data from 2,029 adolescents (51% female) from the TRacking Adolescents’ Individual Lives Survey (TRAILS) were used (*M*_*age*_ [*SD*] at T1 = 11.1 [0.55], T2 = 13.6 [0.52], and T3 = 16.3 [0.70]). Results from stepwise multi-level random-effect models showed that adolescents who experienced an unpleasant move remained stable in internalizing problems, while others decreased over time. Adolescents who moved increased stronger in externalizing problems than adolescents who did not move, independent of whether they experienced the move as unpleasant. Gender and friendship quality before the move did not moderate the relation between residential mobility and internalizing or externalizing problem development. These results emphasize that residential moves in adolescence, especially when experienced as unpleasant, can have long-lasting negative effects on adolescent development.

## Introduction

Adolescence is a turbulent developmental period that is marked by changes in multiple life domains, from physical maturation and identity development to changes in societal roles (Dahl & Gunnar, 2009). For some, adolescence is also a period of increasing internalizing problems (e.g., anxiety, depression, or somatic complaints) or externalizing problems (e.g., aggression, disruptive conduct, or substance use; Polanczyk et al., 2015; Rapee et al., 2019). Internalizing and externalizing problems in adolescence can continue into adulthood and have negative consequences for outcomes in different life domains, such as educational achievement (McLeod et al., 2016), income in adulthood (Veldman et al., 2017), and work performance (Narusyte et al., 2017). Multiple studies linked stressful live events in adolescence to internalizing and externalizing problems (Kim et al., 2003; Leban, 2021). One potential stressful event that has been related to more internalizing and externalizing problems in adolescence is residential mobility (for a review, see Jelleyman & Spencer, 2008; Simsek et al., 2021). However, residential mobility research is mainly based on cross-sectional research designs, which are not able to distinguish between- and within-person effects. Moreover, the effects of residential mobility are likely to differ between individuals or contexts, but it is largely unknown for whom residential mobility is particularly detrimental. To fill these gaps in the literature, the current study has two main aims. First, to examine whether residential mobility in adolescence can aggravate internalizing or externalizing problems, this study investigates the relationship between adolescent residential mobility and internalizing or externalizing problems longitudinally. Second, this study applies a person-centered approach (Laursen & Hoff, 2006) to investigate whether the relation between residential mobility and internalizing or externalizing problem behavior depends on the individual characteristics of subjective moving experience, gender, and friendship quality before the move.

### Residential Mobility and Internalizing and Externalizing Problems

Why would adolescent residential mobility matter for internalizing or externalizing problem development? From a developmental perspective, the multiple co-occurring developmental tasks make adolescence a turbulent developmental period (Steinberg & Silk, 2002). The ontological security theory argues that people have a basic need for continuity, security, and a stable base to return to in order to maintain mental well-being, especially in times that can be threatening or uncontrollable, like the turbulence of adolescence (Giddens, 1991). One’s home is a crucial factor of stability and security (Hiscock et al., 2001). Residential mobility can disrupt this security, leading to feelings of rootlessness or anxiety (Oishi & Talhelm, 2012; Tønnessen et al., 2016). In addition, the social capital theory (Coleman, 1988) assumes that the disruption of social ties following residential mobility can lead to lower well-being. Because of the crucial role of peer networks and friendships for adolescent development and well-being (Brown & Larson, 2009), the disruption of existing friendships and the challenge to find new ones could be a heavy burden on the shoulders of adolescents, leading to increases in internalizing or externalizing behavior problems.

In line with these theories, existing research has repeatedly found a link between residential mobility in childhood, and especially in adolescence, and adolescent internalizing and externalizing problems (for a review, see Cotton, 2016; Simsek et al., 2021). Moves in adolescence were related to internalizing problems in adolescence (Anderson & Leventhal., 2016; Li et al., 2019;), including depression (Björkenstam et al., 2017; Fowler et al., 2015) and mood disorders (Mok et al., 2016). In addition, adolescent residential mobility was related to externalizing problems in adolescence (Flouri et al., 2013; Simsek et al., 2021), including antisocial behavior (Mok et al., 2016), delinquent behavior, and substance use (Brown et al., 2012; Schmidt et al., 2020). One major drawback of these previous studies is that they were based on cross-sectional retrospective data. Due to this methodological limitation, these studies can only reveal whether movers differ significantly from non-movers with regard to the outcome measure, but not whether individuals changed in behavior after a move compared to before. This may be particularly problematic, as some researchers suggest that the relationship between residential mobility and internalizing and externalizing problems might be spurious, driven by selection effects (Gasper et al., 2010). That is, pre-existing differences between mobile and non-mobile families may select families into specific moving behaviors. For example, families with an immigrant background (Kuyvenhoven et al., 2022), lower socioeconomic status (SES; Gasper et al., 2010), or with divorced or single parents are more likely to move than other families (Fomby & Sennott, 2013; Vidal & Baxter, 2018). These family characteristics also correlate with internalizing and externalizing problems in adolescence (Amone-P’Olak et al., 2009) and may thus be confounding factors that explain why adolescents who moved report higher internalizing and externalizing behavior problems than non-moving adolescents (Porter & Vogel, 2014). Supporting this, family socioeconomic status or previous stressful events confounded the relation between residential mobility and adolescent internalizing problems (Flouri et al., 2013; Norford & Medway, 2002). In addition, the relation between residential mobility and adolescent externalizing problems was attenuated after controlling for family instability (Fomby & Sennot, 2013).

Although controlling for confounders is a first step, there might be unobserved factors confounding the relationship between residential mobility and adolescent internalizing and externalizing problems. Longitudinal research distinguishing between-person and within-person differences can largely account for this problem. Between-person differences reveal whether movers differ significantly from non-movers in their internalizing and externalizing problems. With within-person differences, individual behavior before the move is compared to behavior after the move, revealing whether behavior changes within an individual over time. So far, only very few longitudinal studies distinguished between-person from within-person effects in the relation between residential mobility and internalizing and externalizing problems. These studies were all performed in the United States and some cases studied extreme forms of externalizing problems (e.g., delinquent behavior) in high-risk samples (Coley et al., 2013; Vogel et al., 2017). They revealed that adolescents who had moved reported more internalizing or delinquent problems than those who did not move (between-person difference), but there was no within-person increase in internalizing problems or delinquent behavior after the move (Coley et al., 2013; Gasper et al., 2010; Vogel et al., 2017). This suggests a spurious relation between residential mobility and internalizing or delinquent behaviors.

### The Moderating Role of Subjective Moving Experience, Gender, and Friendship Quality

People move for various reasons, from proactive moves to improve their quality of life to reactive moves in response to, mostly, negative life events such as divorce or loss of employment (Coulter & Scott, 2015; Geist & McManus, 2008). Reactive moves are often thought of as stressful moves, with a higher likelihood of negative consequences than proactive moves that improve living circumstances (DeLuca & Jang–Trettien, 2020). However, in most cases the decision to move is made by the parents and the children have to follow, whether they agree with the move or not. Even though a move could mean objective improvement of housing or environment, it could still mean a temporary disruption of stability, friendships, and routines for the adolescent. Thus, even when a move improves living circumstances, it might be experienced as unpleasant and disruptive by the adolescent in the short term. This means that adolescents’ subjective moving experiences are crucial to take into account when one wants to understand the effects of residential mobility on adolescent internalizing or externalizing problems.

Another factor that might affect the effect of residential mobility on internalizing and externalizing problems is gender. Due to hormonal differences, adolescent girls tend to be more sensitive to the negative effects of stressful life events (such as residential mobility) and interpersonal stressors (such as friendship-related issues) than adolescent boys (De Looze et al., 2020; Oldehinkel & Bouma, 2011). Particularly, girls are more likely to respond to such stressful life events with internalizing problems than boys (Matud, 2004). This implies that girls would be more vulnerable to the negative effects of residential mobility and disruption of peer networks after a move. However, there are no studies yet that empirically investigated the moderating role of gender in the relation between residential mobility and internalizing and externalizing problems. Studies that examined the role of gender in other stressful events, such as parental divorce, indeed found that adolescent girls increased stronger in internalizing symptoms after parental divorce than adolescent boys (Oldehinkel et al., 2008).

Finally, given the paramount position of friendship quality for adolescent well-being (Brown & Larson, 2009, Luijten et al., 2021), it is important to study whether friendship quality before the move can moderate the negative effects of residential mobility. On the one hand, following the social capital perspective (Coleman, 1988), moving might be more detrimental for adolescents with high-quality friendships than for adolescents with low-quality friendships before the move, since adolescents with high-quality friendships have to cope with a disruption of their high-quality network. Following this line of reasoning, adolescents with low-quality friendships may have less ‘to lose’ when moving, which implies they might be less negatively affected by a residential move. Moreover, for those with high-quality friendships before the move, it might be more difficult or it may take longer to replace their friendships, which suggests that adolescents with high-quality friendships before the move may (temporarily) be more negatively affected by residential mobility (Barzeva et al., 2022). On the other hand, high-quality friendships can act as a buffer in stressful events (Askeland et al., 2020). Having high-quality friendships before the move may protect adolescents against the negative effects of residential mobility on internalizing and externalizing problems, also because high-quality friendships might be not as easily disrupted by a residential move as low-quality friendships. Moreover, considering having high-quality friendships as an indicator of having the social skills to make friends (Flannery & Smith, 2017), those with high-quality friendships before the move may relatively easily make new friends after the move. Together, these lines of reasoning indicate that having high-quality friendships before the move may moderate the association between moving and internalizing and externalizing problems, either positively or negatively

## Current Study

The impact of residential mobility on adolescent development remains largely unknown, leaving a significant gap in the understanding of which adolescents may be affected by this life event and how. This study aims to fill this gap by investigating whether residential mobility in adolescence is related to internalizing or externalizing problems in adolescence and whether subjective moving experience, gender, and friendship quality before the move moderate this relationship. To answer this research question, a large representative sample of Dutch adolescents from the TRacking Adolescents’ Individual Lives Survey (TRAILS; Ormel et al., 2012) was used. Multiple hypotheses were formulated: First, based on the ontological security theory, it was hypothesized that residential mobility in adolescence is related to an increase in internalizing and externalizing problems in the years after the move (Hypothesis 1a). The effects of residential mobility were expected to fade over time as adolescents get used to their new environments (Hypothesis 1b). Second, it was expected that the negative effects of residential mobility would be stronger for adolescents who experienced the move as unpleasant than for adolescents who did not (Hypothesis 2). Third, the negative effects of residential mobility on internalizing and externalizing problems were expected to be stronger for adolescent girls than for boys (Hypothesis 3). Fourth, based on the social capital theory, high-quality friendships before the move were expected to be a risk factor. This means that the negative effects of residential mobility on internalizing and externalizing problems were expected to be stronger for adolescents with higher-quality friendships before the move (Hypothesis 4a). Conversely, high-quality friendships could also be a protective factor. In that case, the negative effects of residential mobility were expected to be weaker for adolescents with higher-quality friendships than for those with lower-quality friendships (Hypothesis 4b).

## Methods

### Data and Sample Selection

Data for this study originate from the first three waves of the longitudinal cohort study Tracking Adolescents’ Individual Lives Survey (TRAILS; Ormel et al., 2012). The first wave was conducted in 2001 when adolescents were 11 years old and the second and third waves were conducted when adolescents were 13 and 16 years old. Adolescents participating in TRAILS were selected in a two-step selection procedure. First, five municipalities in the northern part of the Netherlands (both rural and urban) were asked to provide a list of all inhabitant adolescents born between 1 October 1989 and 30 September 1990 (first two municipalities) and 1 October 1990 and 30 September 1991 (last three municipalities). Second, all 135 primary schools in these municipalities were asked to participate in the study (122 agreed on participation). School participation was a prerequisite for eligible adolescents and parents to be approached. Parents of 3,145 adolescents were asked to participate. In total, 210 adolescents (6.7%) were excluded due to serious mental or physical health problems or when no Dutch-speaking parent was available (when parents were Turkish or Moroccan, they were interviewed in their own language). When parents agreed to participate, parents, adolescents, and teachers were interviewed. In this study, the adolescent-report data were used. Responders and non-responders in wave 1 did not differ on demographic factors or internalizing and externalizing problems (De Winter et al., 2005). Attrition was slightly higher for males, adolescents with nonwestern immigrant backgrounds, divorced parents, low SES, low IQ, low peer status, low academic achievement, poor physical health, or externalizing problems (Huisman et al., 2008).

For the current study, participants with missing values on moving^1^ (*n* = 141) and control variables (*n* = 38) were excluded. Of the remaining sample, 76% had data on internalizing problems and 78% on externalizing problems in all three waves. Participants with data on only one wave (*n* = 21) were excluded since there was no development to examine. Other missing data were handled with listwise deletion. Analyses were performed on a total sample of 2,029 participants and 6,087 observations.

### Measures

#### Internalizing and externalizing problems

The Dutch version (Verhulst et al., 1996) of the Youth Self Report scale (Achenbach & Rescorla, 2001) was used to measure internalizing and externalizing problems at waves 1, 2, and 3. Adolescents reported these problems in the preceding 6 months on a three-point scale (0 = not or not true, 1 = somewhat true, 2 = very often). The internalizing problem scale consisted of 31 items, including withdrawn/depressed, anxious/depressed, and somatic complaints (α = 0.85; α = 0.88; α = 0.89 for the three waves respectively). An example question is: “I feel worthless or inferior”. The externalizing problem scale consisted of 32 items, including aggressive and delinquent behavior (including substance abuse; α = 0.90; α = 0.85; α = 0.87 for the three waves respectively). An example question is: “I destroy other people’s things”. Total internalizing and externalizing behavior scores were averages of all available items in the subscales, where higher scores indicated more behavior problems.

#### Residential mobility

Adolescent residential mobility was measured at wave 2 with the question: “Have you moved to a different place of residence in the past two years?” (i.e., capturing moves between waves 1 and 2). Answers were dichotomous (1 = yes, 0 = no). To control for the history of residential mobility prior to wave 1, parental reports on past residential moves measured at wave 1 were used as a dichotomous variable (1 = moved, 0 = not moved).

#### Subjective moving experience

The item: “How unpleasant did you find it when you moved to another place of residence?”, with answer categories not unpleasant at all (1), little unpleasant (2), quite unpleasant (3), and very unpleasant (4) was used to measure subjective moving experience. The variable was dichotomized by merging the last three categories (not unpleasant experience = 0, unpleasant experience = 1).

#### Gender

Gender was coded as 0 (girl) and 1 (boy).

#### Friendship quality

The friendship items of the Social Production Functions scale (Nieboer et al., 2005) measured the quality of peer relationships in wave 1. Because this paper focuses on the role of friendship quality before the move, friendship quality measured in the first wave was used. Adolescents reported on nine items about their relation with friends on a five-point scale (1 = never to 5 = always). An example question is: “Many friends help me when something is wrong”. Reversed items were recoded, so that higher scores indicated higher friendship quality (α = 0.90). Friendship quality was standardized with the mean at 0 and a standard deviation of 1.

#### Family characteristics

To control for potential confounding by family characteristics, it was measured at wave 1 whether adolescents lived in a single-parent household (1 = yes, 0 = no) and whether parents had an immigrant background (1 = at least one parent was born abroad, 0 = both parents were born in the Netherlands). In addition, adolescents were asked whether their parents divorced in the past 2 years (1 = yes, 0 = no) or whether they had a newborn sibling in the past 2 years (1 = yes, 0 = no) to control for family changes in the same period as the move. Family socioeconomic status (SES) was measured in wave 1 and constructed by computing the mean of standardized educational level, occupation (using the International Standard Classification for Occupations, Ganzeboom & Treiman, 1996), and income of both father and mother. For the main analyses, SES was included as a continuous variable. Higher scores mean higher SES. When the values of one of the parents were missing, this did not affect the scale significantly (see also Veenstra et al., 2006).

### Analyses

Data were prepared and analyzed in Stata version 17 (StataCorp, 2021). First, to identify potential confounders, Spearman’s correlations were computed between internalizing and externalizing problems at wave 1, residential mobility between waves 1 and 2, gender, friendship quality before the move, SES, immigrant background, divorce between waves 1 and 2, newborn sibling between waves 1 and 2, single-parent family, subjective moving experience, and childhood moves (i.e., before wave 1). Second, stepwise multi-level random-effect models with Maximum Likelihood estimation were run separately for internalizing and externalizing problems. To examine whether observations (level 1) were nested within individuals (level 2), an empty model (Model 0) was estimated. In Model 1, the time variable wave was added to investigate the development of internalizing and externalizing problems over the three waves. To examine whether the development of internalizing and externalizing problems differed between movers and non-movers, Model 2a included the cross-level interaction between wave and move. Model 2b included the control variables gender, SES, immigrant background, family structure, divorce, newborn sibling, and childhood moves in order to control for individual and family factors. The effect of divorce between waves 1 and 2 on internalizing and externalizing problems was time-variant^2^. To distinguish the effects of residential mobility from the effects of a potential co-occurring divorce, the interaction of time*divorce was included as a time-variant control variable.

To examine whether the effects of residential mobility were stronger for those who experienced the move as unpleasant, Model 3 added the cross-level interaction wave*subjective moving experience in the subsample of movers. To investigate whether moving effects differed between girls and boys, Model 4 included the three-way interaction wave*moving*gender. Finally, to examine whether the effects of moving were different for different friendship quality levels, Model 5 included the three-way interaction wave*moving*friendship quality. To investigate potential attrition effects, analyses were performed on a restricted sample of participants who participated in all three waves.

## Results

### Descriptive Statistics

Frequencies and percentages of the sample are presented in Table 1. Of the total sample, 13.4% (*n* = 291) moved between waves 1 and 2. The mean ages in waves 1, 2, and 3 were 11.1 (*SD* = 0.55), 13.6 (*SD* = 0.52), and 16.3 (*SD* = 0.70), respectively. Of those who moved, 52.2% indicated they experienced the move as unpleasant (*n* = 152). Spearman’s correlations were used to identify potential confounding relationships (Table 2). Prior to the move, there were no differences in internalizing or externalizing problems between those who were going to move and those who were not. Gender and friendship quality correlated significantly with internalizing and externalizing problems at wave 1, but not with residential mobility. Single-parent family and childhood moves positively correlated with residential mobility, but not with internalizing or externalizing problems in wave 1. This means that demographic variables could not confound the relationship between residential mobility and internalizing and externalizing problems. Correlations additionally show that adolescents who experienced a divorce or newborn sibling in the past 2 years, those living in a single-parent family, and those who already moved in childhood experienced the move more often as unpleasant. Note that sample sizes are relatively small, so these correlations should be interpreted as explorative. Means and standard deviations of internalizing and externalizing problems are presented in Table 3. There was a decreasing trend in internalizing, but an increasing trend in externalizing problems over the three waves.

**Table 1:** Descriptive statistics of non-movers, movers, and the total sample

	Not moved		Moved		Total	
	*n*	%	*n*	%	*N*	%
Total	1738	85.66	291	14.34	2029	100.00
Gender						
Girl	898	86.02	146	13.98	1044	51.45
Boy	840	85.28	145	14.72	985	48.55
Background						
No immigrant background	1574	85.87	259	14.13	1833	90.34
Immigrant background	164	83.67	32	16.33	196	9.66
Divorce past 2 years						
No	1660	86.59	257	13.41	1917	94.48
Yes	78	69.64	34	30.36*	112	5.52
Newborn sibling past 2 years						
No	1656	86.57	257	13.43	1913	93.32
Yes	100	72.99	37	27.01*	137	6.68
Family structure						
Two-parent	1496	86.57	232	13.43	1728	85.17
Single-parent	242	80.40	59	19.60*	301	14.83
Childhood moves						
No	598	90.88	60	9.12	658	32.43
Yes	1140	83.15	231	16.85*	1371	67.57

*p < 0.05 group difference in likelihood of moving between wave 1 and 2 as analyzed in bivariate logistic regressions (more detailed information is available upon request from the first author)

**Table 2:** Spearman’s rank correlations initial internalizing and externalizing problems

Variables	(1)	(2)	(3)	(4)	(5)	(6)	(7)	(8)	(9)	(10)	(11)	(12)
(1) Internalizing problems wave 1	1.000											
(2) Externalizing problems wave 1	0.531*	1.000										
(3) Moved (vs not moved)	0.019	0.006	1.000									
(4) Boy (vs girl)	−0.117*	0.172*	0.008	1.000								
(5) Friendship quality	−0.152*	−0.216*	−0.027	−0.178*	1.000							
(6) SES	−0.036	−0.019	−0.034	−0.012	0.006	1.000						
(7) Immigrant background (vs non-immigrant)	0.030	−0.015	0.023	−0.019	0.034	−0.140*	1.000					
(8) Divorced (vs not divorced)	−0.017	0.009	0.095*	−0.006	−0.018	−0.075*	−0.063	1.000				
(9) Newborn sibling (vs no newborn sibling)	0.040	0.021	0.094*	−0.006	0.021	−0.097*	0.053*	0.045*	1.000			
(10) Single parent (vs two parents)	0.023	0.029	0.059*	−0.011	0.002	−0.214*	0.084*	0.082*	0.127*	1.000		
(11) Experienced move as unpleasant	0.021	−0.001	0.687*	−0.033	−0.003	−0.02 3	0.024	0.106*	0.102*	0.051*	1.000	
(12) Moved in childhood (vs not moved)	−0.009	−0.020	0.109*	−0.002	0.028	0.016	0.053	0.020	0.061*	0.057*	0.088*	1.000

* = *p* < 0.05

**Table 3:** Internalizing and externalizing problems for non-movers, movers, and the total sample

	Not moved			Moved			Total		
	*M*	SD	*n*	*M*	SD	*n*	*M*	SD	*N*
Internalizing problems									
Wave 1	0.37	0.24	1717	0.37	0.23	281	0.37	0.24	1998
Wave 2	0.33	0.24	1730	0.36	0.26	288	0.33	0.24	2018
Wave 3	0.31	0.24	1381	0.32	0.26	210	0.31	0.25	1591
Externalizing problems									
Wave 1	0.27	0.19	1725	0.27	0.20	286	0.27	0.19	2011
Wave 2	0.28	0.19	1738	0.32	0.21	290	0.29	0.20	2028
Wave 3	0.31	0.21	1396	0.34	0.22	214	0.31	0.21	1610

Scales of internalizing and externalizing problems ranged from 0 to 2

### Residential Mobility and Changes in Internalizing and Externalizing Problems

Results of hierarchical multilevel random effect models are presented in Table 4 (internalizing problems) and Table 5 (externalizing problems). Model 0 showed that observations were nested within individuals and thus that multilevel analyses were required for both internalizing and externalizing problems. Within-person (σ^2^_e_ = 0.03, *SE* = 0.001) and between-person variance (σ^2^_u_ = 0.03, *SE* = 0.001) in internalizing problems were significant. The intra-class correlation (ICC) indicates that 48.4% of the variance in internalizing problems was between persons and 51.6% within persons. For externalizing problems, 45.0% of the variance was between individuals and 55.0% within individuals (σ^2^_e_ = 0.022, *SE* = 0.001; σ^2^_u_ = 0.018, *SE* = 0.001). These results indicate that individual internalizing and externalizing problems changed within individuals over time, but also differed between individuals.

**Table 4:** Hierarchical Multilevel Models of Internalizing Problems predicted by Residential Mobility: Random Effects Models

	Model 0		Model 1		Model 2a		Model 2b		Model 3 SME		Model 4 Gender		Model 5 FQBM	
	*b*	*SE*	*b*	*SE*	*b*	*SE*	*b*	*SE*	*b*	*SE*	*b*	*SE*	*b*	*SE*
Time														
Wave 2			−0.04^**^	0.01	−0.04^**^	0.01	−0.04^**^	0.01	−0.06^*^	0.02	−0.00	0.01	−0.19^**^	0.04
Wave 3			−0.06^**^	0.01	−0.06^**^	0.01	−0.06^**^	0.01	−0.10^**^	0.02	−0.01	0.01	−0.29^**^	0.04
Moved					0.00	0.02	−0.00	0.02			0.00	0.02	−0.04	0.08
Wave 2 *moved					0.03	0.02	0.02	0.02			0.01	0.02	0.02	0.09
Wave 3 *moved					0.00	0.02	0.00	0.02			−0.00	0.02	−0.15	0.09
Boy (vs. girl)							−0.11^**^	0.01	−0.11^**^	0.02			−0.12^**^	0.01
SES							−0.01^*^	0.01	−0.01	0.01	−0.01^*^	0.01	−0.01^*^	0.01
Immigrant background							0.01	0.01	0.00	0.04	0.01	0.01	0.01	0.01
Single-parent family							0.01	0.01	−0.03	0.03	0.01	0.01	0.01	0.01
Divorce last 2 years							−0.02	0.02	−0.01	0.05	−0.02	0.02	−0.03	0.02
Wave 2 *divorce							0.07^**^	0.02	0.09	0.05	0.07^**^	0.02	0.07^**^	0.02
Wave 3 *divorce							0.04	0.03	0.07	0.06	0.03	0.03	0.04	0.03
Newborn sibling							0.03	0.02	0.01	0.03	0.03	0.02	0.03	0.02
Childhood moves							0.01	0.01	0.01	0.03	0.01	0.01	0.01	0.01
Unpleasant moving experience									0.01	0.03				
Wave 2* Unpleasant moving experience									0.06^*^	0.03				
Wave 2* Unpleasant moving experience									0.08^*^	0.03				
Boy (vs. girl)											−0.05^**^	0.01		
Wave 2*boy											−0.08^**^	0.01		
Wave 3*boy											−0.11^**^	0.01		
Moved*boy											−0.01	0.03		
Wave 2*moved*boy											0.02	0.03		
Wave 3*moved*boy											0.01	0.03		
Friendship quality													−0.07^**^	0.01
Wave 2* friendship quality													0.04^**^	0.01
Wave 3* friendship quality													0.06^**^	0.01
Moved* friendship quality													0.01	0.02
Wave 2*moved* friendship quality													0.00	0.02
Wave 3*moved* friendship quality													0.04	0.02
Constant	0.34^**^	0.00	0.37^**^	0.01	0.37^**^	0.01	0.41^**^	0.01	0.41^**^	0.03	0.38^**^	0.01	0.71^**^	0.04
σ_u_	0.17^**^	0.00	0.17^**^	0.00	0.17^**^	0.00	0.16^**^	0.00	0.15^**^	0.01	0.16^**^	0.00	0.16^**^	0.00
σ_e_	0.17^**^	0.00	0.17^**^	0.00	0.17^**^	0.00	0.17^**^	0.00	0.18^**^	0.01	0.17^**^	0.00	0.17^**^	0.00
*N*_obervations_	5607.00		5607.00		5607.00		5607.00		779.00		5607.00		5538.00	
*N*_individuals_	2029.00		2029.00		2029.00		2029.00		291.00		2029.00		2000.00	
aic	−1057.62		−1150.23		−1149.37		−1319.19		−98.18		−1405.00		−1390.99	
bic	−1037.72		−1117.08		−1096.31		−1206.45		−19.00		−1259.11		−1238.74	

Trails waves 1, 2, and 3

σ_u_ = between-person standard deviation, σ_e_ = within-person standard deviation

*SME* subjective moving experience, *FQBM* friendship quality before the move

* = *p* < 0.05, ** = *p* < 0.01

**Table 5:** Hierarchical Multilevel Models of Externalizing Problems predicted by Residential Mobility: Random Effects Models

	Model 0		Model 1		Model 2a		Model 2b		Model 3 SME		Model 4 Gender		Model 5 FQBM	
	*b*	*SE*	*b*	*SE*	*b*	*SE*	*b*	*SE*	*b*	*SE*	*b*	*SE*	*b*	*SE*
Time														
Wave 2			0.02^**^	0.00	0.01^*^	0.01	0.01	0.01	0.04^*^	0.02	0.03^**^	0.01	−0.11^**^	0.03
Wave 3			0.04^**^	0.01	0.04^**^	0.01	0.04^**^	0.01	0.06^**^	0.02	0.06^**^	0.01	−0.14^**^	0.03
Moved					0.00	0.01	−0.00	0.01			−0.01	0.02	0.00	0.07
Wave 2 *moved					0.03^*^	0.01	0.03^*^	0.01			0.04^*^	0.02	0.08	0.07
Wave 3 *moved					0.03^*^	0.01	0.03	0.01			0.03	0.02	0.02	0.08
Boy (vs. girl)							0.04^**^	0.01	0.04^*^	0.02			0.04^**^	0.01
SES							−0.01^*^	0.00	−0.02	0.01	−0.01^*^	0.00	−0.01^*^	0.00
Immigrant background							0.00	0.01	−0.00	0.03	0.00	0.01	0.01	0.01
Single-parent family							0.03^**^	0.01	0.00	0.03	0.03^**^	0.01	0.03^**^	0.01
Divorce last 2 years							−0.00	0.02	−0.01	0.04	0.00	0.02	−0.00	0.02
Wave 2 *divorce							0.04	0.02	0.03	0.04	0.04	0.02	0.04^*^	0.02
Wave 3 *divorce							0.06^*^	0.02	0.05	0.05	0.05^*^	0.02	0.06^*^	0.02
Newborn sibling							0.03^*^	0.01	0.05	0.03	0.03^*^	0.01	0.03	0.01
Childhood moves							0.00	0.01	−0.01	0.02	0.00	0.01	0.00	0.01
Unpleasant moving experience									0.01	0.02				
Wave 2* Unpleasant moving experience									−0.00	0.03				
Wave 2* Unpleasant moving experience									0.01	0.03				
Boy (vs. girl)											0.07^**^	0.01		
Wave 2*boy											−0.05^**^	0.01		
Wave 3*boy											−0.04^**^	0.01		
Moved*boy											0.01	0.03		
Wave 2*moved*boy											−0.02	0.03		
Wave 3*moved*boy											−0.02	0.03		
Friendship quality													−0.06^**^	0.01
Wave 2* friendship quality													0.03^**^	0.01
Wave 3* friendship quality													0.04^**^	0.01
Moved* friendship quality													−0.00	0.02
Wave 2*moved* friendship quality													−0.01	0.02
Wave 3*moved* friendship quality													0.00	0.02
Constant	0.29^**^	0.00	0.27^**^	0.00	0.27^**^	0.00	0.24^**^	0.01	0.25^**^	0.03	0.23^**^	0.01	0.46^**^	0.03
σ_u_	0.14^**^	0.00	0.14^**^	0.00	0.14^**^	0.00	0.13^**^	0.00	0.14^**^	0.01	0.13^**^	0.00	0.13^**^	0.00
σ_e_	0.15^**^	0.00	0.15^**^	0.00	0.15^**^	0.00	0.15^**^	0.00	0.15^**^	0.00	0.15^**^	0.00	0.15^**^	0.00
*N*_obervations_	5649.00		5649.00		5649.00		5649.00		790.00		5649.00		5579.00	
*N*_individuals_	2029.00		2029.00		2029.00		2029.00		291.00		2029.00		2000.00	
aic	−3058.21		−3130.44		−3136.02		−3188.34		−369.01		−3211.41		−3228.68	
bic	−3038.29		−3097.25		−3082.90		−3075.48		−289.59		−3065.35		−3076.27	

Trails waves 1, 2, and 3

σ_u_ = between-person standard deviation, σ_e_ = within-person standard deviation

*SME* subjective moving experience, *FQBM* friendship quality before the move

* = *p* < 0.05, ** = *p* <0.01

**Fig. 1:**
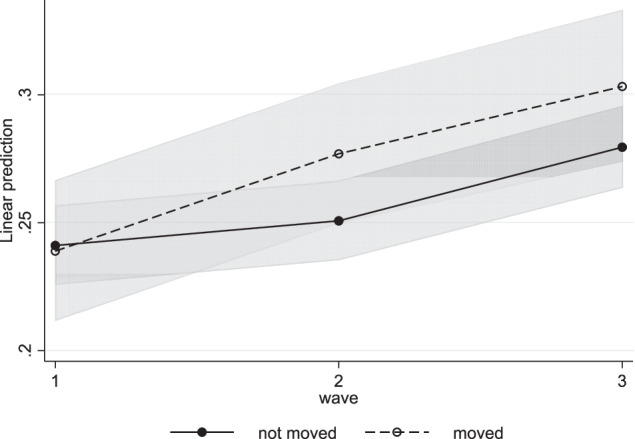
Externalizing problems for moving and non-moving adolescents. Predictive margins of externalizing problem development for movers and non-movers separately, including control variables

In Model 1, the effect of time was added to the total sample. Internalizing problems decreased significantly over the waves (Table 4), whereas externalizing problems increased (Table 5). Model 2a added the cross-level interaction between time and moving, to examine whether and how internalizing and externalizing problems developed differently for movers and non-movers (Table 4). Contrary to hypotheses 1a and 1b, the development of internalizing problems did not differ significantly between movers and non-movers. Externalizing problems however increased significantly stronger between waves 1 and 2 for movers compared to non-movers (Fig. 1), supporting hypothesis 1a. Between waves 2 and 3, externalizing problems increased equally for movers and non-movers, in line with hypothesis 1b. These results hold after controlling for gender, SES, ethnic background, family structure, divorce, newborn sibling, and childhood moves in Model 2b.

### Subjective Moving Experience as Moderator

**Fig. 2:**
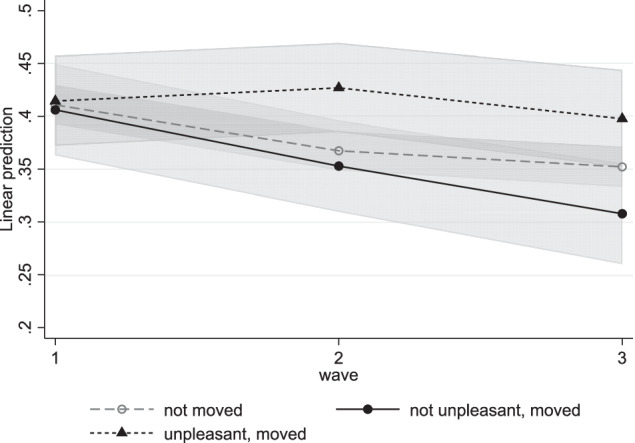
Internalizing Problems for Moving Adolescents by Subjective Moving Experience. Predictive margins of internalizing problem development for moving adolescents who experienced the move as unpleasant and for those who did not separately. The development of non-moving adolescents was added for comparison, but not included in this interaction model

Model 3 added the cross-level interaction between time and subjective moving experience for adolescents who moved (Table 4). In line with hypothesis 2, the interaction between time and subjective moving experience was positive and significant for internalizing problems between waves 1 to 2 (*b* = 0.06, *SE* = 0.03, *p* = 0.040) and between waves 1 to 3 (*b* = 0.09, *SE* = 0.03, *p* = 0.024). Figure 2 shows that all adolescents who moved started with similar levels of internalizing problems, but adolescents who did not experience their move as unpleasant decreased their internalizing problems between waves 1 and 2 (*b* = −0.06, *SE* = 0.02, *p* = 0.014) and between waves 1 and 3 (*b* = −0.10, *SE* = 0.03, *p* < 0.001). However, those who experienced the move as unpleasant showed stable levels of internalizing problems over time (Table 4). This resulted in significantly higher levels of internalizing problems for those who experienced the move as unpleasant compared to those who moved but did not experience this move as unpleasant in wave 2 (*b* = 0.07, *SE* = 0.03, *p* = 0.010). This gap persisted into wave 3 (*b* = 0.09, *SE* = 0.03, *p* = 0.007). For externalizing problems, no significant interaction was found. Externalizing problems increased over time for adolescents who moved, but in contrast to hypothesis 2, this increase was not stronger for those who experienced the move as unpleasant (Table 5).

### Gender as Moderator

**Fig. 3:**
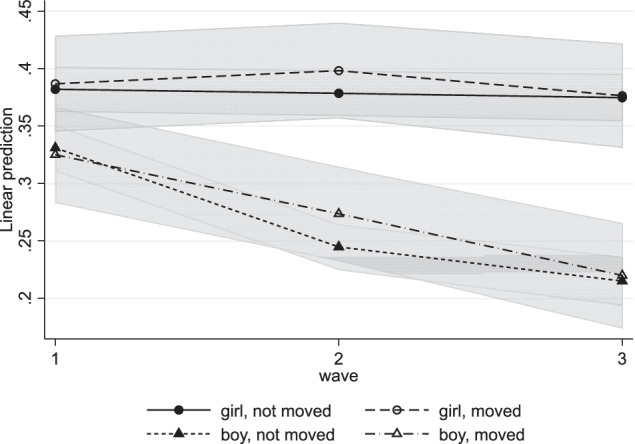
Internalizing Problems by Moving and Gender

**Fig. 4:**
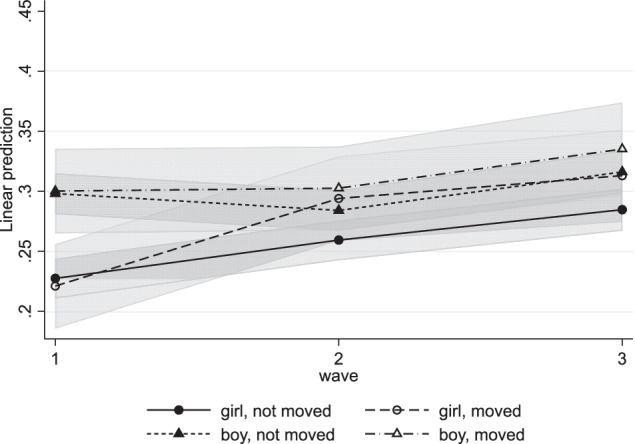
Externalizing Problems by Moving and Gender

To investigate whether the effects of moving on internalizing and externalizing problems differed between boys and girls, three-way cross-level interactions time*moving*gender were added in Model 4. The interactions for internalizing problems were not significant. As shown in Fig. 3, there is a gender difference in the development of internalizing problems, with girls reporting higher and stable levels and boys having lower and decreasing levels of internalizing problems. However, these developments did not differ between those who moved and those who did not. Thus, contrary to hypothesis 3, the effect of residential mobility on internalizing problems did not differ between girls and boys. For externalizing problems, the three-way interactions were not significant either (Table 5). Girls increased significantly more in externalizing problems than boys, but this effect did not depend on residential mobility (Fig. 4). Thus, there were no significant gender differences in the relation between residential mobility and internalizing and externalizing problems.

### Friendship Quality as Moderator

To investigate whether the relationship between residential mobility and internalizing problems was dependent on friendship quality before the move, Model 5 added three-way interaction time*moving*friendship quality. In contrast with hypothesis 4, friendship quality did not moderate the relation between residential mobility and internalizing problems (Table 4). In addition, in contrast to our expectations, friendship quality did not moderate the relation between residential mobility and externalizing problems (Table 5). Specifically, externalizing problems increased for adolescents who moved, and this increase did not depend on friendship quality before the move.

To test whether the three-way interaction between time*moving*friendship quality was non-linear, the interactions were replicated including friendship quality before the move as a categorical variable with low, average, and high friendship quality (0 = 1 standard deviation below the mean or lower, 1 = average, and 2 = 1 standard deviation above the mean or higher). Friendship quality moderated the relation between residential mobility and internalizing problems in a non-linear way. Movers with high friendship quality before the move increased significantly stronger in both internalizing (*b* = 0.08, *SE* = 0.04, *p* = 0.032) and externalizing (*b* = 0.07, *SE* = 0.03, *p* = 0.025) problems between waves 1 and 2 compared to non-moving adolescents with high friendship quality. Residential mobility did not significantly affect internalizing and externalizing problems in adolescents with low or average friendship quality, as the development of internalizing and externalizing problems across the waves showed a similar pattern for movers and non-movers (more detailed results are available upon request of the first author). Because the groups of movers with low and high friendship quality were small (*n* = 56 and *n* = 61, respectively), these results should be interpreted as explorative.

### Sensitivity

To test the robustness of our results, all analyses were performed on a restricted sample of participants who participated in all three waves. Results did not differ between the full sample and restricted sample, except for the effect of subjective moving experience on internalizing problem development. This effect was significant in the total sample but could not reach statistical significance in the restricted sample. Plotting the results provided similar graphs for the restricted sample as for the full sample, indicating similar trends. The lack of significance in the restricted sample is likely due to the small group of movers that experienced the move as unpleasant in this sample (*n* = 101). Overall, sensitivity analyses indicate that attrition did not seriously affect the results (detailed results are available upon request from the first author).

## Discussion

Many adolescents face the challenge of residential mobility in their lives, but whether this life event affects adolescent development, and for whom this is the case, is largely unknown. Up to date, research has been mainly correlational and variable-oriented. This study addressed this gap by examining the relationship between residential mobility in adolescence and internalizing and externalizing problems longitudinally, with a focus on individual differences. Results showed that, in general, internalizing problems were decreasing over time. However, adolescents who experienced an unpleasant move did not decrease in internalizing problems, leaving them with higher levels of internalizing problems even years after the move. No differences in responses to residential mobility were found between boys and girls. There was no linear effect of friendship quality before the move on the relation between residential mobility and internalizing and externalizing problems. In addition, adolescents who moved increased significantly more in externalizing problems than non-moving adolescents. This effect was not dependent on subjective moving experience, gender, and friendship quality. Since there were no differences in internalizing or externalizing problems between movers and non-movers before the move and relationships remained significant after controlling for individual and family characteristics, there was no evidence for selection as suggested by Gasper and colleagues (2010). These results indicate that residential mobility in adolescence is a life event that has the potential to increase internalizing and externalizing problems.

### Internalizing Problems

Adolescents who moved, but who did not experience this move as unpleasant, showed a similar development of internalizing problems as non-movers. However, adolescents who experienced an unpleasant move remained stable in their internalizing problems. Although not every move is experienced as unpleasant, the moves that are experienced as such, have negative effects on adolescents internalizing problem development. This result is concerning for several reasons. Firstly, the majority of the adolescents who moved experienced the move as unpleasant, which means these experiences are not an exception. Secondly, the disparity in internalizing problems between moving and non-moving adolescents persisted into wave 3, spanning years after the move. Thirdly, explorative analyses showed that adolescents experienced a move more often as unpleasant when they experienced other stressful life events in the same 2 years, such as parental divorce, or when they were living in a single-parent family. This points towards cumulative disadvantage. Previous literature supports this trend, showing that moves that are commonly perceived as stressful and unpleasant often follow other challenging life events, such as divorce, housing problems, or unemployment. These types of moves are particularly prevalent in socioeconomically disadvantaged families (DeLuca & Jang–Trettien, 2020). When residential mobility fails to alleviate adolescents’ internalizing problems, residential mobility may contribute to cumulative disadvantage and exacerbate social inequality in later life. It should be noted that not all moves were experienced as unpleasant. One important finding of this study is that these moves did not affect internalizing problem development negatively. The reason for the move or the distance moved might be important predictors of subjective moving experience. For example, in short-distance moves, adolescents might be able to stay in contact with their old friends, which makes the move a less unpleasant experience. However, moves are generally of short distance in the Netherlands (Kuyvenhoven et al., 2022) and still, the majority of this Dutch sample experienced the move as unpleasant. This highlights the complexity of residential mobility experiences. Future research is encouraged to dive deeper into predictors of children and adolescents’ subjective moving experiences.

No gender differences were found in internalizing responses to residential mobility. In line with previous research (Gutman & McMaster, 2020; Oldehinkel & Bouma, 2011), girls, independent of whether they moved or not, reported higher and stable levels of internalizing problems in adolescence, whereas boys reported lower and decreasing levels. The finding that girls did not respond to residential mobility with an increase in internalizing problems might be explained by a ceiling effect: girls reported relatively high levels at the first wave, which means there is less room to increase further after a residential move.

The effect of residential mobility did not depend on friendship quality before the move. Two competing hypotheses were formulated, but none of these were supported by the data. From a social capital perspective, it was expected that adolescents with high-quality friendships before the move would show more negative effects of residential mobility than those with lower-quality friendships before the move. From a buffer perspective, it was expected that high-quality friendships before the move could protect against the negative effects of the move and that social skills would enable one to make new friends quickly. The fact that no interaction was found, could indicate that these mechanisms have canceled each other out. Adolescents may hold on to old high-quality friendships until they have formed new friendships. Moreover, there may be a short-term effect, which could not be captured due to the 2-year interval of the current study. Adolescents may feel lonely right after the move when they do not have friends yet (Tønnessen et al., 2016), which could result in a temporary peak in internalizing problems (Nangle et al., 2003; Vernberg et al., 2006). However, most adolescents can make new friends within a year (Vernberg et al., 2006). As soon as they have settled down and made new friends, problems are likely to decrease (La Greca & Harrison, 2005; Waldrip et al., 2008). Finally, the explorative analyses suggest that the effect of friendship quality before the move is not linear and that a move is only detrimental for those with high-quality friendships before the move, in line with the social capital perspective. Note that the average of friendship quality was already relatively high (4 on a scale from 1 to 5). This means that those who scored one standard deviation above the mean or higher had extraordinary high-quality friendships. Due to the small sample size, this result should be interpreted with caution. However, this finding can provide relevant input for future research. In addition, future research is encouraged to study internalizing problem development directly after the move and monitor this with short time intervals.

### Externalizing Problems

Although increases in externalizing problems were normative in this study sample, the increase in externalizing problems between waves 1 and 2 was stronger for movers than for non-movers. This was in line with the hypothesis. Residential mobility was related to increasing externalizing problems for all movers and did not depend on subjective moving experience, gender, or friendship quality before the move. It should be noted that also for externalizing problems, explorative results hint towards a non-linear effect in which moving negatively affected those with high friendship quality before the move even stronger than other movers. However, these results should be interpreted as exploratory and replicated with larger representative samples.

One mechanism that could explain the stronger increase in externalizing problems in moving adolescents could be found in the social networks of these adolescents. Movers are more likely to be involved in deviant peer networks than non-movers (Haynie & South, 2005; Haynie et al., 2006), which explaines movers’ higher levels of externalizing problems (Schmidt et al., 2020). It is important to note that non-moving adolescents increased in externalizing problems as well, but slower and more steadily between waves 1 and 3. This could be explained by the fact that alcohol use and smoking were part of the externalizing problem scale. The use of these substances is commonly initiated in adolescence (Gray & Squeglia, 2018). These substances were legally available in the Netherlands from age 16^3^ at the time of the study, which is the average age of participants at wave 3. One could expect a general increase in externalizing problems between waves 2 and 3 due to the use of these substances. The results of this study suggest that adolescents who moved may have started earlier with these substances than those who did not move, potentially driven by deviant peer networks. Future research is encouraged to test this mechanism longitudinally. Moreover, it is worth studying whether results are different in contexts with a different legal age of substance use. In 2014, the legal age for alcohol use and smoking in the Netherlands increased to 18. Future research should therefore replicate this study in the current Dutch context and other contexts.

### Strengths and Limitations

The longitudinal nature of the TRAILS data and the large representative sample enabled to study both within and between-person differences. With that, normative age effects, moving effects, and confounding effects could be separated, with a focus on the role of subjective moving experience, gender, and friendship quality. As such, the current study adds important insights to research on residential mobility effects. However, some limitations have to be considered. First, detailed information about the move was lacking. That is, the exact date of the move between waves 1 and 2 was unknown as well as the amount of moves within this period and the distance of the move. From a life course perspective (Elder & Shanahan, 2006), moving timing, frequency, and distance matter for its outcomes (Vogel et al., 2017; Widdowson & Siennick, 2021). The Netherlands is a small country, in which relatively short-distance moves are overrepresented (Kuyvenhoven et al., 2022). Moreover, only moves between waves 1 and 2 were included, which means between ages 11 and 13. Hence, the results of the current study may be unique to Dutch early adolescents and therefore not generalizable to residential mobility in other contexts and age ranges. Second, the current study focused on friendship quality before the move. Friendship quality however is subject to change over time. To examine whether and how a move relates to changes in friendship quality after the move, and how friendships after the move relate to internalizing and externalizing problems, more longitudinal research with a social network focus is needed. Third, data for this study was collected between 2001 and 2005. Nowadays, social media have changed the scene of social networks and friendships. Social media can help to maintain friends from before the move (Steinfield et al., 2008), which possibly changes the experience of moving. Fourth, in TRAILS, participants could only report their gender as male or female. Adolescence is a period of identity exploration, including gender identity (Watson et al., 2020). Although beyond the scope of this article, it might be that especially for adolescents struggling with their gender identity, a stable home base is crucial. This could be an interesting alley for future research. Finally, residential mobility is likely to co-occur with parental divorce (Fomby & Sennott, 2013). However, in our study, it was unknown whether parental divorce and residential mobility were linked events, or whether they happened independently. Since only 34 adolescents experienced both residential mobility and divorce within the same 2 years, it was impossible to distinguish between the effects of residential mobility and divorce in this study.

## Conclusion

Residential mobility is a common experience over the life course and may not be seen as a negative life event per se. Past research emphasized that adolescence is a turbulent developmental period in which the instability of residential mobility can be detrimental. To exclude the possibility that associations between residential mobility and negative outcomes are due to selection, longitudinal research is needed. This longitudinal study showed that residential mobility in adolescence could be an unpleasant experience with detrimental and long-lasting effects on adolescent development. Moves experienced as unpleasant, which was the case for more than half of the moves, had negative effects on internalizing problem development. Indeed, residential mobility was related to within-person increases in externalizing problems even when the move was not experienced as unpleasant. These longitudinal associations were not explained by selection. This study emphasizes that adolescence is a vulnerable period for the challenges that come along with residential mobility. Because internalizing and externalizing problems in adolescence can have negative effects on development into adulthood, residential mobility has the potential to affect individuals negatively over their life courses. This study can inform parents planning to move, but also teachers and practitioners to realize that moving is a serious challenge for adolescents and that they may need support during and after a residential move.

## Footnotes

1 Missing data on moves between waves 1 and 2 was more likely in boys, participants with immigrant backgrounds, lower SES, and single-parent families.

2 Internalizing problems increased significantly right after divorce (*b* = 0.07, *SE* = 0.02, *p* = 0.002), but this effect vanished into wave 3. Externalizing problems increased right after divorce (*b* = 0.04, *SE* = 0.02, *p* = 0.032). between waves 2 and 3, adolescents who experienced a divorce between waves 1 and 2 did not increase stronger in externalizing problems than those who did not experience a divorce.

3 Since 2014, the legal age for these substances has been increased to 18 years.
